# Editorial: Mental health of healthcare professionals

**DOI:** 10.3389/fpsyg.2022.1093569

**Published:** 2022-12-09

**Authors:** Feng Jiang, Huanzhong Liu, Yi-lang Tang

**Affiliations:** ^1^School of International and Public Affairs, Shanghai Jiao Tong University, Shanghai, China; ^2^Institute of Healthy Yangtze River Delta, Shanghai Jiao Tong University, Shanghai, China; ^3^Department of Psychiatry, Chaohu Hospital of Anhui Medical University, Hefei, China; ^4^Department of Psychiatry, School of Mental Health and Psychological Sciences, Anhui Medical University, Hefei, China; ^5^Department of Psychiatry, Anhui Psychiatric Center, Hefei, China; ^6^Department of Psychiatry and Behavioral Sciences, Emory University, Atlanta, GA, United States; ^7^Substance Abuse Treatment Program, Atlanta Veterans Affairs Medical Center, Decatur, GA, United States

**Keywords:** mental health, healthcare professionals (HCPs), stakeholder, governance, intervention

According to the World Health Organization, mental health is defined as a state of mental wellbeing that enables individuals to cope with stresses, realize their abilities, work well, learn well, and contribute to society (World Health Organization., 2003). Compared to the general population, healthcare professionals are more likely to encounter mental health problems, and these symptoms not only affect the wellbeing of those affected but also often negatively impact the healthcare quality and workforce sustainability (Windover et al., 2018; Anderson et al., 2021; Hodkinson et al., 2022). On the other hand, mental health is considered an integral and essential component of health, and it is also a type of health human capital (Hooker, 2021), which can be invested and maintained (Lim et al., 2018; Stein and Sridhar, 2019; Wang et al., 2021). Recent studies suggested that, in the setting of the COVID-19 pandemic, the overall mental health status of healthcare professionals has worsened due to multiple factors, including heavier workload, fear of COVID-19, psychological distress, and other environmental factors (Labrague and de Los Santos, 2021). Therefore, the mental health issue of healthcare professionals deserves more attention from researchers and policymakers (Spoorthy et al., 2020).

As the guest editors for the Special Issue on the Mental Health of Healthcare Professionals, we would like to highlight five research articles here. These articles involved different perspectives and different samples, including healthcare professionals themselves, family members, hospital management, policymakers, and the public.

In healthcare workers, Li et al. demonstrated that one-fourth of psychiatrists in China experienced depressive and anxious symptoms during the COVID-19 pandemic, calling for urgent action. Shi et al. showed that primary healthcare workers reported high levels of anxiety and depression symptoms in China during the pandemic. They also demonstrated that social support and resilience significantly mediate the relationship between work stress and anxiety/depression, highlighting the roles of social support (resources) and individual coping skills (resilience) and possible targets for interventions. Actions should be taken by physicians and other stakeholders, such as the hospitals' management, healthcare policymakers, patients, family members, and others.

In family members, Alimoradi et al. found that healthcare professionals coped communally within their families, especially with their spouses, in dealing with work-related stress. As family members are often the primary support, any intervention strategies should consider involving families.

In hospital management and peers, Rizzi et al. demonstrated that individual support sessions were a protective and supportive factor for healthcare professionals' mental health. The psychological support program was recommended for all COVID-19 patients' care units. This research suggested that hospital management and peers can take effective psychological actions to improve the health human capital of healthcare workers.

In health policymakers, Pacutova et al. indicated that pandemic management was strongly associated with the psychological responses of healthcare workers. As health policymakers usually handle pandemic management, the effects are often widespread and systemic. Even before the COVID-19 pandemic, studies had shown that organizational system factors affected the mental health of health professionals (Hall and Friedman, 2013).

In light of this Research Topic's findings, awareness of mental health issues among healthcare professionals should be emphasized. Government, healthcare systems, and healthcare organizations need to create and ensure the infrastructure and resources to support healthcare professionals. Specific actions may vary, but common interventions include having regular surveys and assessments of the stress and mental health symptoms, adjusting their workload, and ensuring time for them to recover (Dang et al., 2020).

The collection of articles in this Issue is an important start. More research is needed, especially interventional studies focusing on the effectiveness of different programs and studies involving interventions by various stakeholders. Studies examing the role of institutional factors and individual coping mechanisms may also inform the development of more targeted interventions.

## Author contributions

FJ, HL, and Y-LT made substantial contributions to the study design. FJ and HL collected data. FJ analyzed the data, interpreted the analysis results, and completed the manuscripts. HL and Y-LT contributed to critical revision of the manuscript. All authors have read and approved the published version of the manuscript.
